# How to keep patients and staff safe from accidental SARS-CoV-2 exposure in the emergency room: Lessons from South Korea’s explosive COVID-19 outbreak

**DOI:** 10.1017/ice.2020.376

**Published:** 2020-07-30

**Authors:** Yun Jeong Kim, Jae Young Choe, Ki Tae Kwon, Soyoon Hwang, Gyu-Seog Choi, Jin Ho Sohn, Jong Kun Kim, In Hwan Yeo, Yeon Joo Cho, Ji Yeon Ham, Kyung Eun Song, Nan Young Lee

**Affiliations:** 1 Department of Emergency Medicine, School of Medicine, Kyungpook National University, Daegu, Korea; 2 Division of Infectious Diseases, Department of Internal Medicine, School of Medicine, Kyungpook National University, Daegu, Korea; 3 Department of Infection Control, Kyungpook National University Chilgok Hospital, Daegu, Korea; 4 Colorectal Cancer Center, Kyungpook National University Chilgok Hospital, School of Medicine, Kyungpook National University, Daegu, Korea; 5 Department of Otolaryngology-Head and Neck Surgery, School of Medicine, Kyungpook National University, Daegu, Korea; 6 Department of Clinical Pathology, School of Medicine, Kyungpook National University, Daegu, Korea

## Abstract

**Objectives:**

We report our experience with an emergency room (ER) shutdown related to an accidental exposure to a patient with coronavirus disease 2019 (COVID-19) who had not been isolated.

**Setting:**

A 635-bed, tertiary-care hospital in Daegu, South Korea.

**Methods:**

To prevent nosocomial transmission of the disease, we subsequently isolated patients with suspected symptoms, relevant radiographic findings, or epidemiology. Severe acute respiratory coronavirus 2 (SARS-CoV-2) reverse-transcriptase polymerase chain reaction assays (RT-PCR) were performed for most patients requiring hospitalization. A universal mask policy and comprehensive use of personal protective equipment (PPE) were implemented. We analyzed effects of these interventions.

**Results:**

From the pre-shutdown period (February 10–25, 2020) to the post-shutdown period (February 28 to March 16, 2020), the mean hourly turnaround time decreased from 23:31 ±6:43 hours to 9:27 ±3:41 hours (P < .001). As a result, the proportion of the patients tested increased from 5.8% (N=1,037) to 64.6% (N=690) (P < .001) and the average number of tests per day increased from 3.8±4.3 to 24.7±5.0 (P < .001). All 23 patients with COVID-19 in the post-shutdown period were isolated in the ER without any problematic accidental exposure or nosocomial transmission. After the shutdown, several metrics increased. The median duration of stay in the ER among hospitalized patients increased from 4:30 hours (interquartile range [IQR], 2:17–9:48) to 14:33 hours (IQR, 6:55–24:50) (P < .001). Rates of intensive care unit admissions increased from 1.4% to 2.9% (P = .023), and mortality increased from 0.9% to 3.0% (P = .001).

**Conclusions:**

Problematic accidental exposure and nosocomial transmission of COVID-19 can be successfully prevented through active isolation and surveillance policies and comprehensive PPE use despite longer ER stays and the presence of more severely ill patients during a severe COVID-19 outbreak.

On March 11, 2020, the World Health Organization (WHO) declared the coronavirus disease 2019 (COVID-19) a global pandemic.^1^ The first patient in South Korea was reported on January 19, 2020.^2^ Since the 31st Korean case, who was the first in Daegu, was diagnosed on February 18, 2020, the number of COVID-19 patients increased explosively because of a cluster infection among a religious group called Shincheonji, which accounted for ~70% of the Daegu cases.^3^ As of March 14, 2020, the number of confirmed patients in the Daegu region accounted for ~74 % of all of the Korean cases (Fig. 1).^4^ To cope with this major epidemic crisis, Daegu was designated a special disaster area on March 15, 2020. Many emergency centers in Daegu were consecutively and repeatedly closed, and medical staff on duty and inpatients were quarantined because of accidental exposure to a COVID-19 patient who had not been isolated.^5^ Our emergency room (ER), which has ~30,000 patient visits annually, is a regional emergency center designated by the Ministry of Health and Welfare and 1 of 6 major ERs in Daegu. As of 2018, 13.5% of all ER patients in Daegu city had visited our ER.^6^

**Fig. 1.**
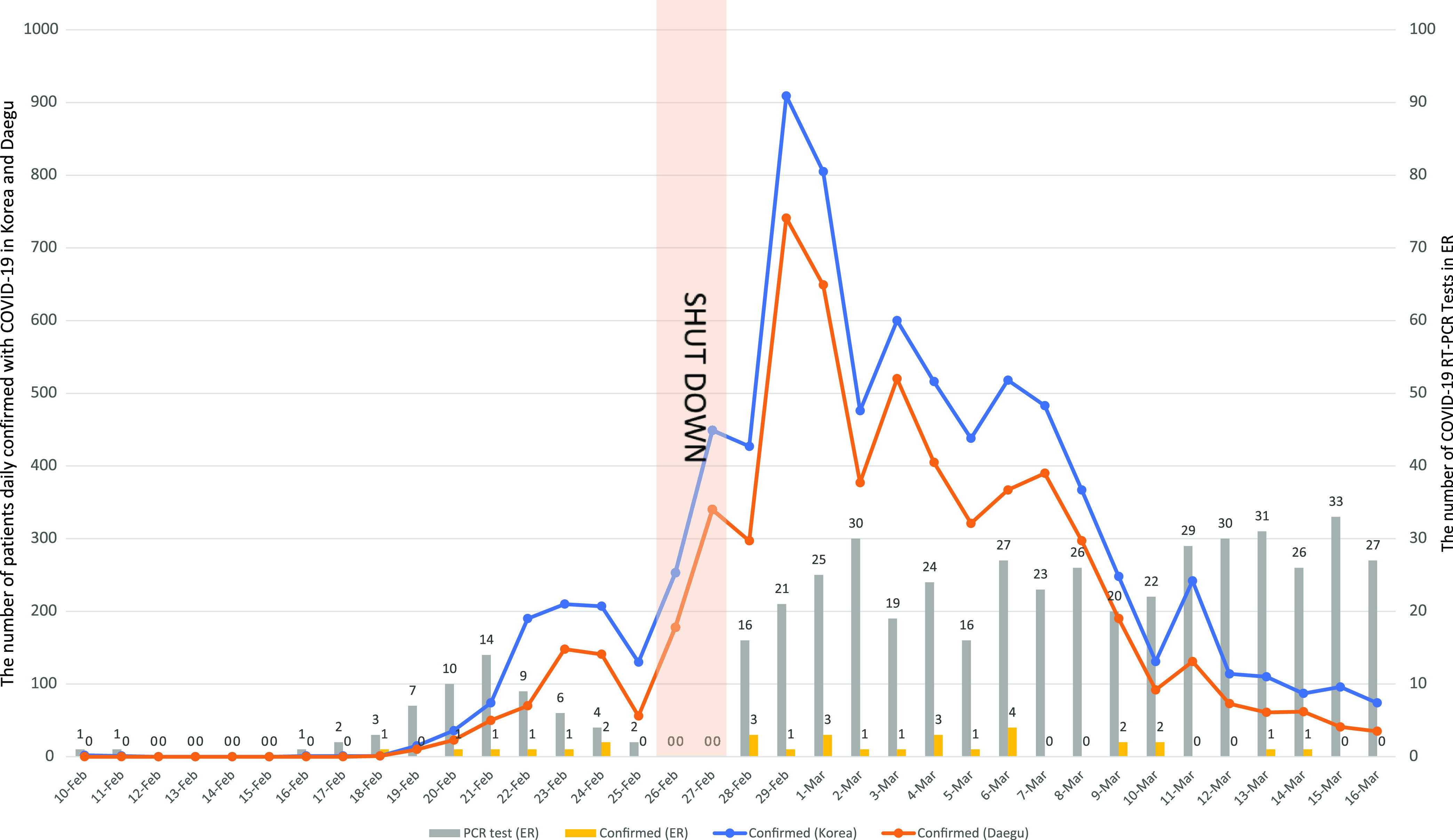
The daily number of patients confirmed with COVID-19 in South Korea and Daegu city and the daily number of SARS-CoV-2 reverse transcriptase-polymerase chain reaction (RT-PCR) and patients with positive results in our emergency room (ER). The daily number of patients confirmed with COVID-19 in South Korea (blue line) and Daegu city (orange line) had reached the peak just after our ER shutdown. The daily number (gray bars) of SARS-CoV-2 RT-PCR and positive results (yellow bars) in our ER increased from the pre-shutdown period to the post-shutdown period.

On February 23, 2020, a 77-year-old male patient visited our ER in an ambulance. He presented with gradual deterioration of mental status, cough, sputum production, and vomiting for 3 days. He was not isolated and was closely monitored in the ER for 31 hours until a severe acute respiratory syndrome coronavirus-2 (SARS-CoV-2) reverse-transcriptase polymerase chain reaction (RT-PCR) test was performed. Subsequently, his wife and he were confirmed to have COVID-19. Among a total of 110 persons (47 patients or guardians and 63 healthcare workers [HCWs]) who were shown to have had contact with them by epidemiological investigation and closed-circuit television (CCTV) monitoring, we determined that 5 people (1 patient and 4 HCWs) needed to be quarantined for 14 days because of inappropriate personal protective equipment (PPE). The ER was closed for 55 hours from 3:50 p.m. on February 25 to 10:30 p.m. on February 27, 2020, while we investigated the close contacts, decontaminated the area, and established new protocols to ensure the safety of the ER.

Protecting HCWs and patients from SARS-CoV-2 while maintaining functional emergency medical care were critical in responding properly to this outbreak.^7^ We implemented new interventions, including active isolation, surveillance, and comprehensive use of PPE in the ER, to prevent recurrence of an ER shutdown and nosocomial transmission of COVID-19. We performed this research to analyze the effects of our interventions during this outbreak.

## Materials and methods

### Study population and design

**Fig. 2.**
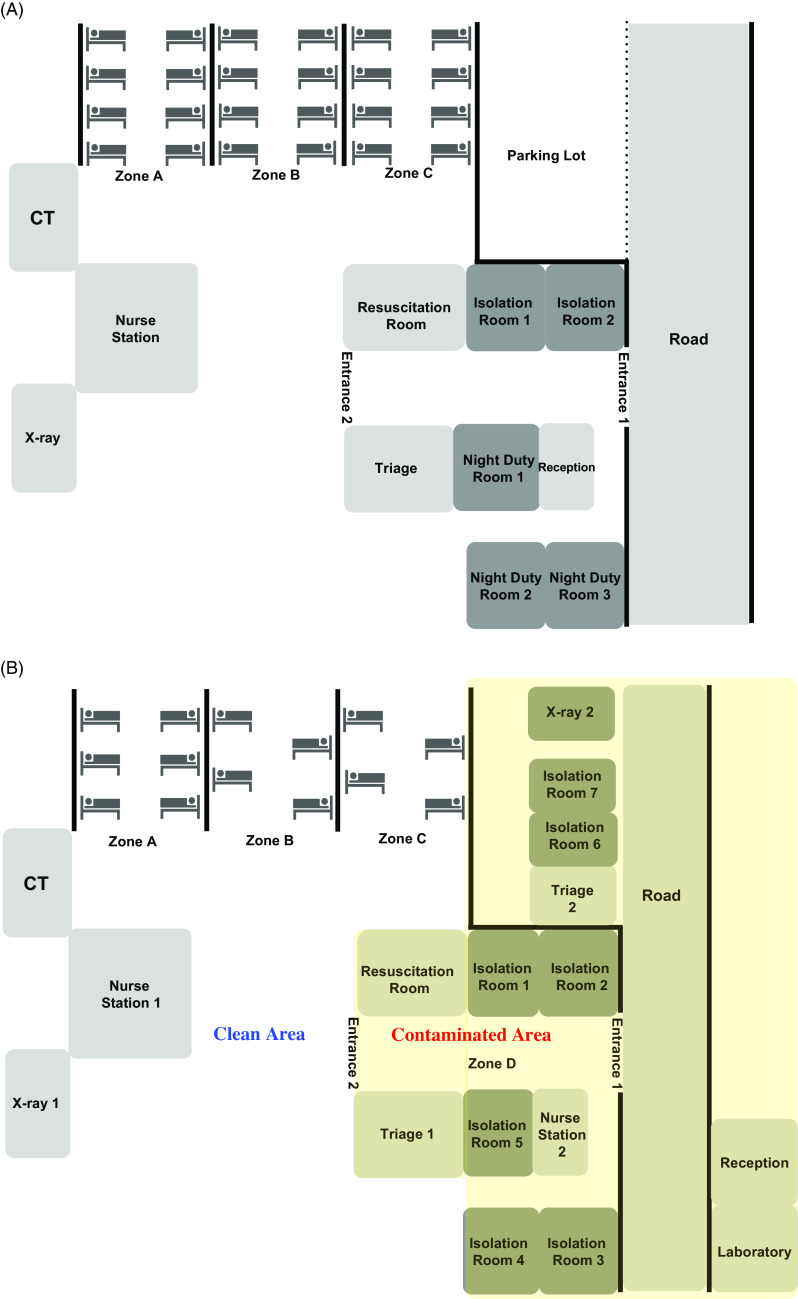
Schematic illustrations of the emergency room structure changes between the pre-shutdown period and the post-shutdown period. (A) The structure of the emergency room (ER) in the pre-shutdown period. Before ER shutdown, there were 24 beds in 3 zones (A, B and C) and 2 nonairborne infection isolation rooms between entrance 1 and entrance 2. The 16 beds for adult patients were divided into zone A and B according to the severity of illness, and zone C contained 8 beds for children. The interbed distance was 1.5 m. (B) The structure of ER in the post-shutdown period. After the ER shutdown, we designated the clean area (blue letters) and the contaminated area (red letters) separated by entrance 2. We set up a triage including a reception area, a laboratory, a chest x-ray area, and a resuscitation room (isolation room 6 or 7) outside the ER using intermodal containers. We built airborne infection prevention systems in the isolation rooms 1, 2, 3, 4, 6 and 7 and x-ray 2 and laboratory rooms using mobile negative-air machines. We reduced the number of beds in zones A, B, and C to 14 and widened the interbed distance to 2 m. High-resolution closed-circuit televisions and portable patient monitors were installed in all of the isolation rooms to monitor vital signs, level of consciousness, and patient movement.

This cross-sectional, observational study was conducted in a 635-bed, tertiary-care, academic hospital in Daegu, South Korea, from February 10 to March 16, 2020. The medical records for all patients visiting the ER during the study period were retrospectively reviewed. After the ER shutdown, we implemented following interventions in the ER: (1) Triage facilities were set up outside the ER (Fig. 2). (2) SARS-CoV-2 RT-PCR testing and chest X-ray were performed outside the ER for most patients who needed to be hospitalized, and these patients were admitted after their COVID-19 status was established. (3) Respiratory samples were obtained in the contaminated area (Fig. 2B) using drive-through or walk-through testing access for patients in stable condition. (4) Patients with respiratory symptoms, fever, abnormal chest x-ray findings, or any epidemiologic relevance to COVID-19 were isolated. (5) A portable negative-pressure isolation chamber was employed for COVID-19 patients and for patients whose COVID-19 status had not been identified but who needed to be moved inside the hospital beyond the ER. (6) A universal mask policy and a comprehensive use of PPE were established. (7) The number of doctors on duty was increased from 8 to 11 and from 23 to 34 for nurses. (8) Real-time communications were established between members of the COVID-19 patient management task force.

For this analysis, we defined the pre-shutdown period as February 10–25, 2020, and the post-shutdown period as February 28 to March 16, 2020. We compared the patient outcomes and durations of ER stay from both periods.

### SARS-CoV-2 RT-PCR

Before the ER shutdown, SARS-CoV-2 RT-PCR was performed by an outside laboratory; after the ER shutdown, it was performed in our laboratory in the hospital. We expanded the regular working shifts of laboratory personnel from the usual 3 shifts to 4 shifts to shorten the turnaround time from sampling to obtaining a result. RNA was extracted from clinical samples with an automated nucleic acid extraction platform Libex (Xian Tianlong Science &Technology, Xi’an, China). SARS-CoV-2 was detected by RT-PCR using a PowerChekTM 2019 nCoV Real-Time PCR Kit (KogeneBiotech, Seoul, Korea) and a CFX96 real-time PCR detection system (Bio-Rad, Berkeley, CA). The statistics for these RT-PCR tests were analyzed, including the total number of tests, average number of tests per day, and turnaround time of tests in the ER between the pre-shutdown period and the post-shutdown period. This study was exempt from review by the institutional review board of the Kyungpook National University Chilgok Hospital (no. KNUCH 2020-03-034).

### Statistical analysis

Continuous variables were expressed as the means ± standard deviation or median (IQR) and were compared using the Student *t* test or the Mann–Whitney U test. Categorical variables were compared with the Pearson χ^2^ test or the Fisher exact test. The time lengths are expressed as HH:MM (ie, hours and minutes). All tests of significance were 2-tailed; *P* ≤ .05 was considered significant. The results were analyzed using SPSS version 21.0 software (IBM, Armonk, NY).

## Results

### COVID-19 RT-PCR test

In total, 1,727 patients were treated in the ER during entire study period (pre-shutdown, n = 1,037; post-shutdown, n = 690) (Table 1). The proportions of the patients in whom SARS-CoV-2 RT-PCR was performed increased from 5.8% to 64.6% (*P* < .001), and the average number of tests per day increased from 3.8±4.3 to 24.7±5.0 (*P* < .001) from the pre-shutdown period to the post-shutdown period (Table 1) (Fig. 1). Among the 690 patients in the post-shutdown period, 245 (35.4%) patients were not tested because they had already been tested (n = 85); they were discharged directly from the ER after asymptomatic short ER stays (n = 153). Also, 6 patients died in the ER after short ER stays. The mean turnaround time decreased from 23:31 ±6:43 hours to 9:27±3:41 hours (*P* < .001) from the pre-shutdown period to the post-shutdown period.

**Table 1.** Changes in General Characteristics and Outcomes Before and After the Shutdown Period

Variables	Before the Shutdown (2/10/20–2/25/20)	After the Shutdown (2/28/20–3/16/20)	*P* Value
Total no. of patients	1,037	690	
**Gender, no. (%)**			.115
Male	543 (52.4)	388 (56.2)	
Female	494 (47.6)	302 (43.8)	
Age, mean y ±SD	44.0±27.6	52.0±26.3	<.001
**Visit type, no. (%)**			<.001
Direct visit	664 (64.0)	399 (57.8)	
119 rescue	162 (15.6)	176 (25.6)	
Transfer	178 (17.2)	79 (11.4)	
Outpatient	32 (3.1)	31 (4.5)	
Others	1 (0.1)	5 (0.7)	
**KTAS, no. (%)**			.157
Level 1	12 (1.2)	12 (1.7)	
Level 2	25 (2.4)	17 (2.5)	
Level 3	361 (34.8)	210 (30.4)	
Level 4	622 (60.0)	445 (64.5)	
Level 5	17 (1.6)	6 (0.9)	
**Performance of RT-PCR**			
Total no. (%)	60 (5.8)	445 (64.6)	<.001
Average no. per day (mean±SD)	3.8±4.3	24.7±5.0	<.001
Turnaround time (mean±SD)	23:31±6:43	9:27±3:41	<.001
**No. of RT-PCR results, no. (%)**			
Positive	7 (11.7)	23 (5.2)	
Negative	53 (88.3)	422 (94.8)	
**Outcome**			.004
Admission to GW (%)	268 (25.8)	196 (28.4)	.239
Admission to ICU (%)	14 (1.4)	20 (2.9)	.023
Transfer to other hospital (%)	13 (1.3)	13 (1.9)	.292
Discharge (%)	733 (70.6)	440 (63.8)	.003
Death (DOA) (%)	9 (1) (0.9)	21 (0) (3.0)	.001
**Median duration of ER stay (IQR)**			
Total cases	2:52 (1:21–5:25)	2:55 (1:06–11:05)	.012
Admission to GW	4:44 (2:33–10:00)	17:18 (7:34–25:21)	.000
Admission to ICU	1:41 (0:59–2:56)	8:38 (1:50–14:52)	.033
Transfer to other hospital	3:01 (1:19–12:00)	10:44 (3:48–23:59)	.038
Discharge	2:28 (1:08–4:12)	1:51 (0:50–3:47)	.002
Death (including DOA)	2:08 (0:57–11:43)	3:08 (1:23–15:26)	.512

Note. SD, standard deviation; KTAS, Korean triage and acuity scale; level 1 (resuscitation), level 2 (emergency), level 3 (urgency), level 4 (less urgency), level 5 (nonurgent); RT-PCR, reverse-transcriptase polymerase chain reaction; GW, general ward; ICU, intensive care unit; DOA, death on arrival; ER, emergency room; IQR, interquartile range.

### Screening and monitoring for nosocomial spread

From February 10 to June 14, a total of 9,177 SARS-CoV-2 RT-PCR tests were performed in our hospital. These included 934 tests for HCWs who had symptoms or any accidental exposure to the COVID-19 patients or who were taking care of COVID-19 patients. Also, 3,585 RT-PCR tests were performed for all ER patients who were hospitalized; 641 RT-PCR tests were performed for inpatients who had symptoms or were quarantined; 1,033 tests were preoperative screening tests; 508 tests were performed for preadmission screening; and 1,782 tests were performed for outpatients. During the outbreak, tests for all HCWs taking care of COVID-19 patients had been routinely performed every 2–4 weeks. In addition, all HCWs, inpatients, and their guardians were monitored daily for their symptoms and had screening tests any time they had symptoms. Through those tests and symptom monitoring, no evidence of person-to-person transmission of SARS-CoV-2 was detected in our hospital from February 10 to June 14.

### Outcomes and durations of ER stays

The number of patients (7 versus 23) confirmed with COVID-19 in the ER increased from the pre-shutdown period to the post-shutdown period (Table 1) (Fig. 1). Among 7 patients confirmed in the pre-shutdown period, 3 patients were admitted to the COVID-19 general care ward, and 4 patients were diagnosed after discharge. In total, 23 COVID-19 patients in the post-shutdown period were isolated in the ER without any problematic accidental exposure and nosocomial transmission. Among them, 10 patients were admitted to the COVID-19 general care ward, 2 patients were admitted to the COVID-19 intensive care unit (ICU), 6 patients were discharged from ER, 3 patients were transferred to other hospitals, and 2 patients who came to the ER in cardiac arrest died and were confirmed positive for COVID-19 posthumously.

The rates of ICU admission (1.4% vs 2.9%, *P* = .023) and mortality (0.9% vs 3.0%; *P* = .001) in the ER increased from the pre-shutdown period to the post-shutdown period (Table 1). Among 9 deceased patients in the pre-shutdown period, 2 patients died after CPR, 3 patients in cardiac arrest died after CPR, and 3 patients died with a do-not-resuscitate (DNR) order. Among 21 deceased patients in the post-shutdown period, 3 patients died after CPR, 8 patients in cardiac arrest died after CPR, and 10 patients died with a DNR order. The 30-day mortality rates among patients admitted to the ICU were not different between the pre-shutdown period and the post-shutdown period: 21.4% (3 of 14) versus 30.0% (6 of 20) (*P* = .577).

The median duration of stay in the ER among hospitalized (general care ward and ICU) patients increased between the pre-shutdown period and the post-shutdown period: 4:30 hours (IQR, 2:17–9:48) versus 14:33 hours (IQR, 6:55–24:50) (*P* < .001). The median duration of stay outside the ER for tests and waiting in the post-shutdown period was 00:44 hours (IQR, 00:17–01:33).

## Discussion

In 2015, South Korea experienced the largest outbreak (186 cases and 38 deaths) of Middle East respiratory syndrome (MERS) outside the Middle East because of massive transmissions from a single, nonisolated patient in an overcrowded ER.^8,9^ This experience caused hospitals in the city of Daegu, which had the first large outbreak of COVID-19 outside China, to respond actively and promptly to the accidental exposure to COVID-19 patients who were not identified for isolation at triage in the ER. In Daegu, 40 temporary ER closures took place, and 6 level-1 or level-2 ERs were shut down 27 times for 769 hours from February 18 to March 26, 2020.^5^ To prevent ER shutdown and nosocomial transmission of COVID-19, many ERs in Daegu revised triage procedures and performed active surveillance and isolation and implemented a universal mask policy and comprehensive use of PPE, similar to our hospital. Consequently, these ERs could operate successfully, even amid a severe COVID-19 outbreak.^5,10^ However, performing triage procedures, testing (laboratory and chest x-ray), and resuscitation outside the ER can increase the duration of stay in the ER and can affect patient outcomes. In fact, overcrowding and long duration of stay in the ER in general hospitals have been a constant problem in Korea. According to the 2015 nationwide survey of ERs, the average duration of stay among 414 ERs in Korea was 6 hours and 45 minutes; the average duration of stay at 20 ERs listed in the order of long stay was 14 hours.^11^ Durations were becoming shorter through much effort but became longer again in the COVID-19 outbreak. The rates of ICU admission and mortality were higher after the interventions were implemented. The patients who came to the ER in cardiac arrest and died after CPR and those who died with DNR order comprised the majority of mortality cases. Therefore, we suspect think that patients with severe conditions could not come to the ER as easily as before because of the saturation of healthcare facilities associated with the COVID-19 outbreak in Daegu, or they were reluctant to come to the ER promptly for fear of being infected with COVID-19. For example, 3 COVID-19 patients in Daegu died at home while waiting for hospitalization.^10^

Early identification and rapid isolation of patients with COVID-19 are crucial to interrupting the spread of this virus.^12,13^ The World Health Organization (WHO) also emphasized that countries need to implement strong measures to detect and achieve laboratory confirmation of their cases early.^14^ In Korea, the Ministry of Food and Drug Safety urgently approved a diagnostic kit for SARS-CoV-2 RT-PCR and required certified private hospitals to use that kit beginning in February 2020.^15^ The high level of test performance made it possible for us to test most patients to be hospitalized and for these patients to wait in the isolation room until the test results were obtained. When they needed to be moved inside the hospital for emergency operations or procedures before test results were obtained, we used a portable negative-pressure isolation chamber and comprehensive PPE. We previously reported on a patient undergoing appendectomy in a negative-pressure operating room with medical personnel wearing comprehensive PPE and including a powered air-purifying respirator.^16^ He had a positive SARS-CoV-2 result after surgery but did not cause any nosocomial transmission of the virus. The drive-through screening system, which was first implemented at our hospital on February 23, 2020, was of great help in speeding up safe respiratory sample collection.^17^

SARS-CoV-2 transmission occurs mainly through respiratory droplets and contact, and airborne transmission may be possible during aerosol-generating procedures (AGPs). In this context, the WHO currently recommends droplet and contact precautions for suspected or confirmed COVID-19 patients and airborne precautions for AGPs.^18^ However, appropriate selection and use of respiratory PPE during the COVID-19 crisis remains controversial.^19^ The Korean Centers for Disease Control and Prevention (KCDC) recommended airborne and contact precautions in any situation involving contact with a suspected or confirmed patient, based on the experience of the 2015 MERS outbreak.^20^ The KCDC initially recommended coveralls with shoe covers and double gloves for contact precautions; eye shield, face shield, and goggles for eye protection; N95 respirators or equivalent for respiratory protection; and powered air-purifying respirators when AGPs are performed.^20^ Long-sleeved, water-resistant gowns and KF94 masks are recommended in the revision of previous recommendations. Following this KCDC guideline, we strengthened the level of the required PPEs in the ER to ensure safety in the events of accidental SARS-CoV-2 exposure. We think the strengthened PPE and universal mask policies played a crucial role in protecting HCWs and patients and guardians from accidental exposure to SARS-CoV-2 in the ER. Although PPE was difficult to obtain in the early stages of this outbreak, similar to the situation in other large cities, the supply was never exhausted. The Korean government and local city authorities controlled the consumption and supply of this critical element of care.^10^ Healthcare facilities and HCWs had the highest priority for obtaining PPE. The role of the government and local city authorities was crucial for controlling the supply and demand of PPE during the outbreak.

The ER, which serves as a gatekeeper for hospitals, is expected to be the area most exposed to SARS-CoV-2. If healthcare facilities fail to organize an effective system for screening, isolating, and testing suspected cases, an increased number of patients and confusion in the ER can turn an ER into the epicenter of a hospital-associated outbreak.^21,22^ The value of intermodal containers used for extra space outside the ER (Fig. 2B) and mobile negative-air machines used in the AIIRs was demonstrated in Korea during the MERS outbreak.^21^ The temporary AIIRs in our ICU using mobile negative-air machines has played a crucial role in managing critically ill COVID-19 patients.^23^ However, intermodal containers and mobile negative-air machines are only temporary equipment. Conventional or mobile telephone communication in the contaminated area was used as much as possible to reduce contact between HCWs and patients. Telemedicine can be useful for improving infection control during the COVID-19 pandemic.^17,24,25^ To smooth the flow of patients, key personnel from the various departments (eg, administration, infectious diseases, respiratory diseases, emergency medicine, COVID-19 general care and ICU nursing teams, and the infection control team) conducted real-time communication using a mobile messaging application to assess the availability of beds, patient acceptance capabilities, and hospitalization process. The integrated response between our team representative and the out-of-hospital emergency system operated by the local government was critical in managing COVID-19 patients properly and preventing accidental SARS-CoV-2 exposure in each ER.

This study has several limitations. First, this study describes the experience of only 1 hospital, and the results may not be generalizable. However, our successful experience could be modified as a suitable model for ER operation in other areas during the COVID-19 crisis. We have provided detailed information for the measures we implemented. Second, this study is a retrospective, observational study. Because multiple interventions were implemented simultaneously, it is difficult to clearly determine which intervention worked significantly. However, a controlled experimental trial was not realistically possible during this swift-moving outbreak.

In conclusion, problematic accidental exposure and nosocomial transmission of the COVID-19 can be successfully prevented through active isolation and surveillance polices and comprehensive PPE use despite longer ER stays and the presence of more severely ill patients during a COVID-19 outbreak.
